# Revealing the local crystallinity of single silicon core–shell nanowires using tip-enhanced Raman spectroscopy

**DOI:** 10.3762/bjnano.11.99

**Published:** 2020-07-31

**Authors:** Marius van den Berg, Ardeshir Moeinian, Arne Kobald, Yu-Ting Chen, Anke Horneber, Steffen Strehle, Alfred J Meixner, Dai Zhang

**Affiliations:** 1 Institute of Physical and Theoretical Chemistry, Eberhard Karls University of Tübingen, Auf der Morgenstelle 15, Tübingen, Germany; 2 Center for Light-Matter Interaction, Sensors & Analytics (LISA+), Eberhard Karls University of Tübingen, Auf der Morgenstelle 15, Tübingen, Germany; 3 Institute of Electronic Devices and Circuits, Ulm University, Albert-Einstein-Allee 45, Ulm, Germany; 4 Institute of Micro- and Nanotechnology, Technische Universität Ilmenau, Max-Planck-Ring 12, Ilmenau, Germany

**Keywords:** core–shell nanowires, local crystallinity, polarization angle-resolved spectroscopy, silicon, tip-enhanced Raman spectroscopy

## Abstract

Tip-enhanced Raman spectroscopy is combined with polarization angle-resolved spectroscopy to investigate the nanometer-scale structural properties of core–shell silicon nanowires (crystalline Si core and amorphous Si shell), which were synthesized by platinum-catalyzed vapor–liquid–solid growth and silicon overcoating by thermal chemical vapor deposition. Local changes in the fraction of crystallinity in these silicon nanowires are characterized at an optical resolution of about 300 nm. Furthermore, we are able to resolve the variations in the intensity ratios of the Raman peaks of crystalline Si and amorphous Si by applying tip-enhanced Raman spectroscopy, at sample positions being 8 nm apart. The local crystallinity revealed using confocal Raman spectroscopy and tip-enhanced Raman spectroscopy agrees well with the high-resolution transmission electron microscopy images. Additionally, the polarizations of Raman scattering and the photoluminescence signal from the tip–sample nanogap are explored by combining polarization angle-resolved emission spectroscopy with tip-enhanced optical spectroscopy. Our work demonstrates the significant potential of resolving local structural properties of Si nanomaterials at the sub-10 nanometer scale using tip-enhanced Raman techniques.

## Introduction

The properties of silicon are long standing topics of various investigations because silicon is still the most widely used semiconductor material for a broad range of micro- and nano-electromechanical systems, microelectronics, and photovoltaics [1–2]. Silicon nanostructures, such as bottom-up-grown nanowires [3], were also synthesized serving as multifunctional platforms for field-effect transistors [4–6], photovoltaic devices [7–10] and miniaturized chemical sensors [5,11–12]. A key element for many of those devices are high-quality nanometer-scale semiconductor junctions, such as pn-junctions that ensure the intended electronic functionality of such nanometer-scale building blocks. A rational and well-established synthesis strategy for the creation of complex silicon nanostructures is metal-catalyzed vapor–liquid–solid (VLS) nanowire growth [13]. VLS nanowire growth belongs to the gas-phase synthesis procedures, similar to chemical vapor deposition (CVD), and enables direct nanowire growth in a bottom-up manner. The nanowire composition, in particular the doping concentration, can be controlled by an adequate adjustment of the synthesis gas mixture, e.g., by setting the SiH_4_/B_2_H_6_ ratio during the synthesis of boron-doped silicon nanowires (SiNWs). A rational strategy to obtain radial homo- and heterojunctions is to overcoat the as-grown nanowires within the same reaction chamber by implementing a conventional CVD process (e.g., thermal SiH_4_-CVD) yielding core–shell nanowires [14]. Although ideal epitaxial growth is frequently assumed, the crystallinity of the shell is intimately linked to multiple process parameters and, thus, subject to local variations at the nanometer scale [15]. Control and knowledge of the crystal state of core–shell nanowires are important to rationally design, understand and control the optical and electronic behavior of nanowire building blocks. Hence, there is an inherent need for non-destructive characterization techniques that are able to elucidate the local crystallinity.

Raman spectroscopy is such a type of non-destructive characterization techniques and has become a popular method to investigate structural properties of silicon samples [16–17]. Mizoguchi et al. [18] and Hopkins et al. [19] utilized it to show the influence of stress on the crystal lattice orientation angles and to determine the degree of surface roughness. Kolb et al. measured the lattice orientational change due to laser-induced temperature variation [20]. Furthermore, crystalline (c-Si) and amorphous (a-Si) Si show different Raman peaks, which can be used to determine the fraction of crystallinity and bond-angle distortion [21–22]. For example, Nikolenko et al. [23] investigated the local structure and phases of silicon by measuring its Raman peaks along a silicon wire prepared under high pressure. They found a shift of the transverse optical (TO) phonon peak along the wire, which could be attributed to a non-uniform growth of the nanowire and different crystalline phases. Agbo et al. showed that polarized excitation Raman spectroscopy is useful to distinguish hydrogenated nano-crystalline silicon films (nc-Si) from a-Si and c-Si areas [24].

Although, Raman spectroscopy is an overall powerful tool to characterize the material properties of Si, this technique requires still an improvement regarding the sensitivity and the diffraction-limited optical resolution. Thus, a high-resolution technique that reveals both, the structural and the optical properties at the nanometer scale is needed to study the fraction of crystalline phases and defects within the SiNWs. Tip-enhanced Raman spectroscopy (TERS) has distinguished itself as a powerful characterization technique, which allows to obtain both the morphology and the so-called chemical “finger print” information simultaneously with a resolution of a few nanometers. The key element of this technique is a sharp tip resembling a nanometer-scale antenna. This nanoantenna is typically made by chemical etching of a thin Ag or Au wire or by evaporating a Ag or Au thin film on AFM tips. The tip works like an optical antenna when it is brought as close as a few nanometers to the sample surface and when it is illuminated with a tightly focused laser beam. The near field localized at the tip apex enhances the optical field in the tip–sample gap by several orders of magnitude and simultaneously directs the emitted photons from the gap into the far field for detection. With recent demonstrations of a spatial resolution even at the angstrom level [25], TERS has made real chemical nanospectroscopy possible [26–28].

In this work, the structural properties of VLS-grown core–shell SiNWs are investigated using both confocal Raman spectroscopy and TERS. Notably, the silicon core is single crystalline while the shell should be amorphous to nanocrystalline, depending on the synthesis parameters. Hence, these nanowires resemble ideal objects to study local crystallinity variations at the sub-10 nanometer scale using TERS. Furthermore, polarization angle-resolved spectroscopy is for the first time combined with TERS, in order to reveal the different polarizations of Raman scattering and the photoluminescence from the tip–sample nanogap.

## Results and Discussion

### Silicon nanowire core–shell morphology

**Figure 1:**
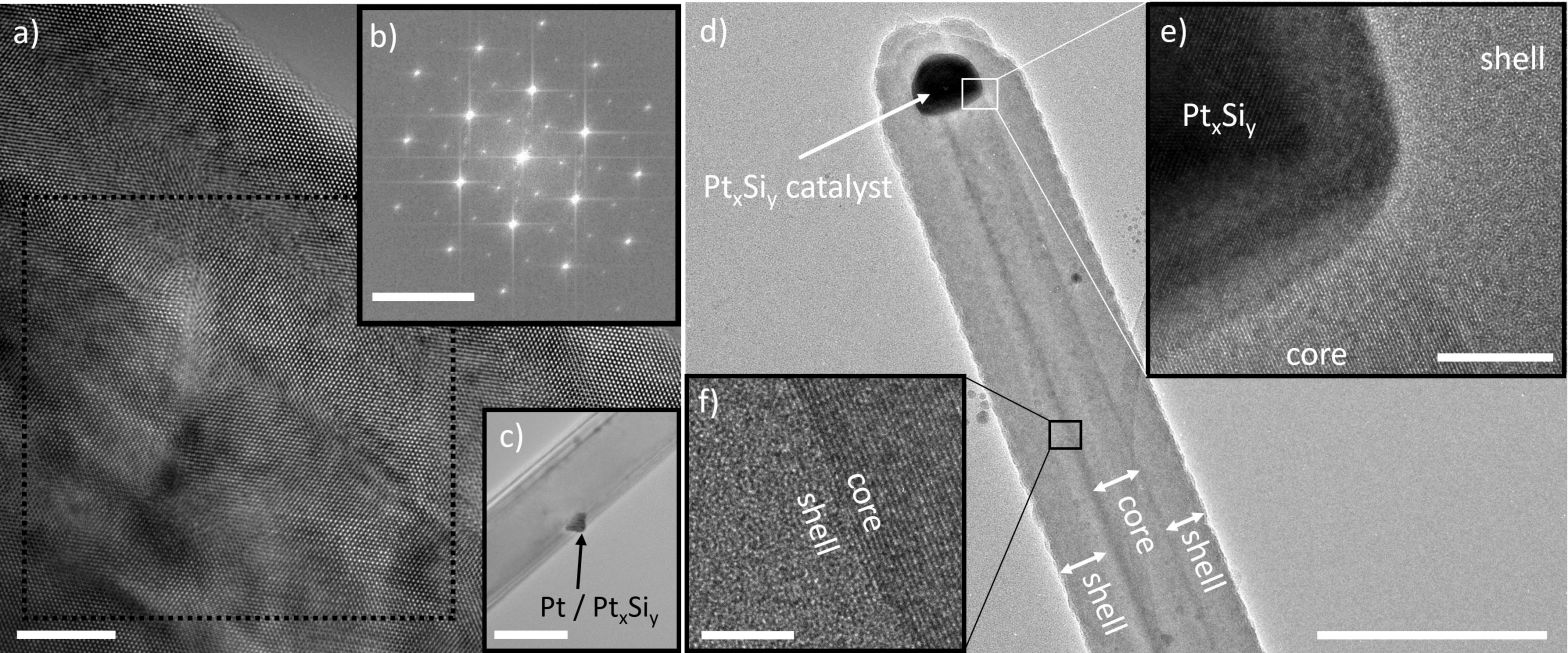
a) High-resolution TEM image of a segment of a SiNW obtained through Pt-catalyzed growth that exhibits several defects and differently oriented crystalline grains. b) The fast Fourier transformation image of the indicated area in panel a. c) TEM image showing a migrated metal particle on a SiNW. d) TEM image of a Pt-catalyzed core–shell SiNW showing the morphology of the junction between SiNW core and shell. e) Pt silicide catalyst and the shell region. f) Illustration of the interface between the SiNW core and the shell. Scale bars are for a) 5 nm, b) 5 nm^−1^, c) 100 nm, d) 200 nm, e) 10 nm and f) 5 nm.

In accordance with the VLS synthesis method (see Experimental section), the utilized Pt catalyst, or finally Pt*_x_*Si*_y_*, remains at the tip of the nanowire during growth. However, it is worth mentioning that other mechanisms of Pt-catalyzed growth of nanowires were also previously observed [29]. The overall morphology of the SiNWs was investigated by transmission electron microscopy (TEM). The high-resolution TEM investigation of the core area indicates that the SiNWs are mainly single crystalline. However, in some areas along the nanowire axis defects are present as well (Figure 1a). The diffraction pattern of this part of the SiNW (Figure 1b) shows more than one reflection, which indicates that the structure of the SiNW consists presumably of segments or grains with different orientation. Furthermore, catalyst migration along the SiNW backbone was observed in some cases (Figure 1c). Although the SiNWs grown by the VLS mechanism possess are crystalline, the silicon shells deposited onto the nanowires by thermal CVD (here using a temperature of 520 °C) can be amorphous [15]. Figure 1d illustrates such a core–shell SiNW with a distinct contrast in core–shell morphology. As it can be seen in Figure 1e and Figure 1f, the core region of the nanowire appears single crystalline and the crystal planes end abruptly at the core–shell interface. The shell region of the nanowire exhibits an amorphous morphology.

### Confocal optical microscopy of silicon nanowires

**Figure 2:**
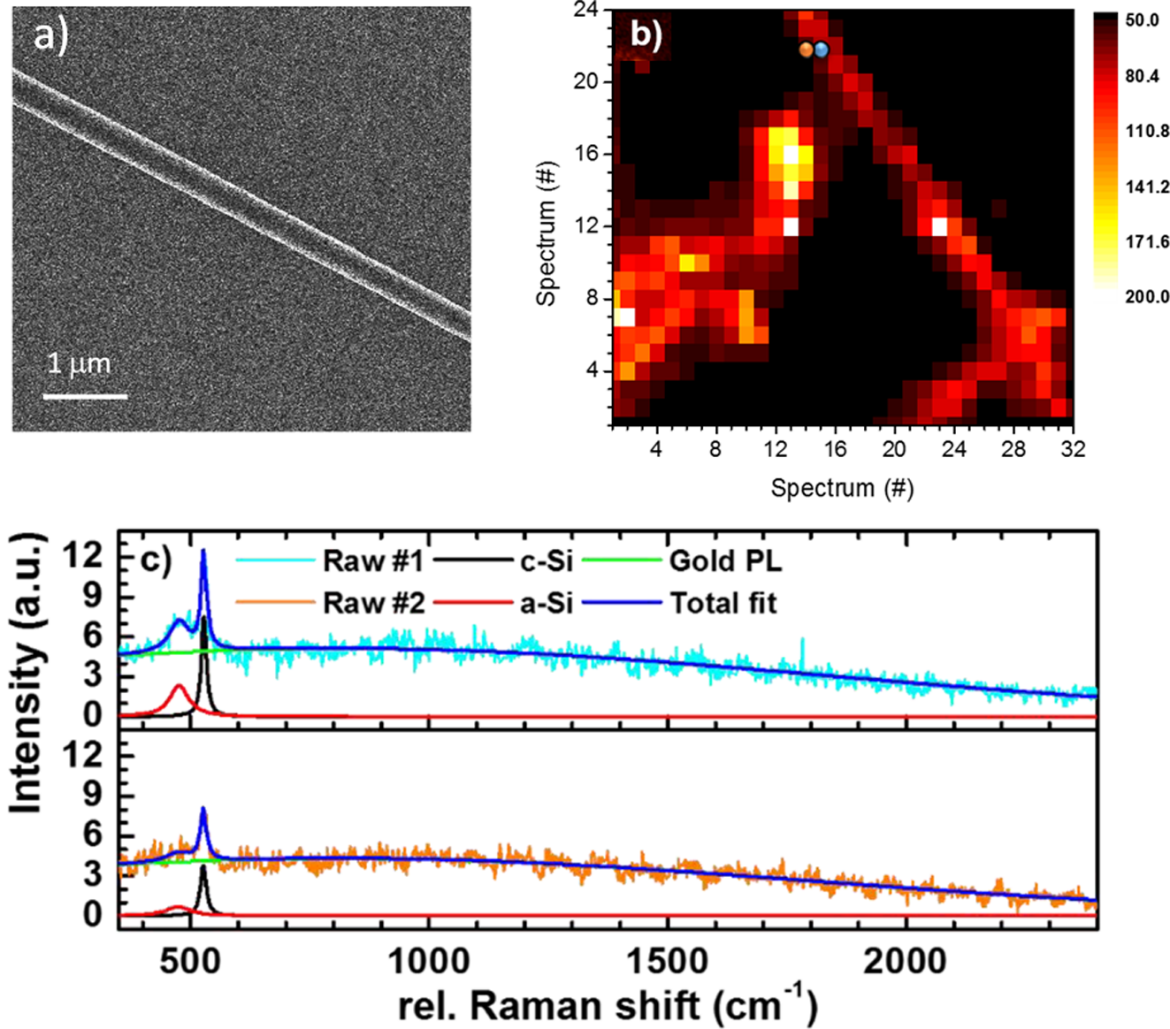
a) Representative helium ion microscopy image of a SiNW, which is supported on a Au-coated Si wafer. b) Hyperspectral image of the sample surface. 32 × 24 spectra are taken from a 20 × 20 µm^2^ area. The color scale is based on the integrated intensity of each taken spectrum. c) Two example spectra taken from the positions marked by the orange and the blue dot in panel b. The original data is plotted in light-blue and orange. The Raman peaks of crystalline and amorphous silicon are fitted using Lorentzian functions, which are indicated by the black and the red line, respectively. The total fit for both spectra is indicated by the dark-blue line. Furthermore, the spectral background is fitted (green line) using a Gaussian function. The background results from photoluminescence of the Au film. Excitation source: 636.3 nm continuous-wave diode laser.

As the first step, core–shell SiNWs grown with a platinum catalyst [29] are characterized using a custom-built confocal optical microscope. Figure 2a shows the representative geometry of these SiNWs, which were characterized using a helium ion microscope. In Figure 2b a hyper-spectral image composed of 32 × 24 spectra taken from an area of 20 × 20 µm^2^ is shown, in which bundles of SiNWs can be seen. In Figure 2c two spectra taken from the positions marked with orange and blue points are given. The light blue and orange lines show the raw spectra, which are composed of a broad photoluminescence continuum emitted from the underlying Au thin film and sharp Raman peaks. For further analysis, these spectral features are fitted using Lorentzian functions for the Raman peaks and a Gaussian function for the photoluminescence peak. The first-order transverse optical mode of c-Si (F_2g_) leads to a sharp Raman peak at 520 cm^−1^; whilst a-Si is detected by a broad band centered at 495 cm^−1^ [28]. A thin oxide layer that covers the SiNWs, causes a broad Raman peak at 480 cm^−1^ and significantly influences the shape of the F_2g_ peak leading to a broadening and a stronger baseline of this peak [30–31]. In order to quantitatively evaluate the local structural composition, the a-Si and c-Si Raman peak intensities are therefore determined by integrating the total Raman intensities in the spectral ranges of 460–500 cm^−1^ for a-Si, and of 514–532 cm^−1^ for c-Si, after subtracting the PL background. To calculate the crystalline fraction (*f*_c_) we use the model of Smit et al. [32] as shown in Equation 1. The areas of the Raman peaks of the c-Si and a-Si are used as the corresponding intensities (*I*_c-Si_ and *I*_a-Si_) for the calculation of crystalline fraction (*f*_c_).

[1] $$f_{\text{c}}=\frac{I_{\text{c-Si}}}{\left(I_{\text{c-Si}}+0.8I_{\text{a-Si}}\right)}.$$

For the spots marked orange and blue Figure 2b, the corresponding spectra are shown in Figure 2c. Values of *f*_c_ = 0.45 and *f*_c_ = 0.57 were calculated, respectively. A further confirmation of the lower crystallinity in the spot marked by the orange dot comes from the full width at the half maximum (FWHM) of the Raman peaks. Both a-Si and c-Si peaks are significantly broader, 25% and 13%, respectively, at the location marked by the orange dot. These results agree well with the morphology revealed in the high-resolution TEM images (Figure 1c) since the sample position with the orange dot is located at the perimeter of the SiNW, where the a-Si shell dominates.

**Figure 3:**
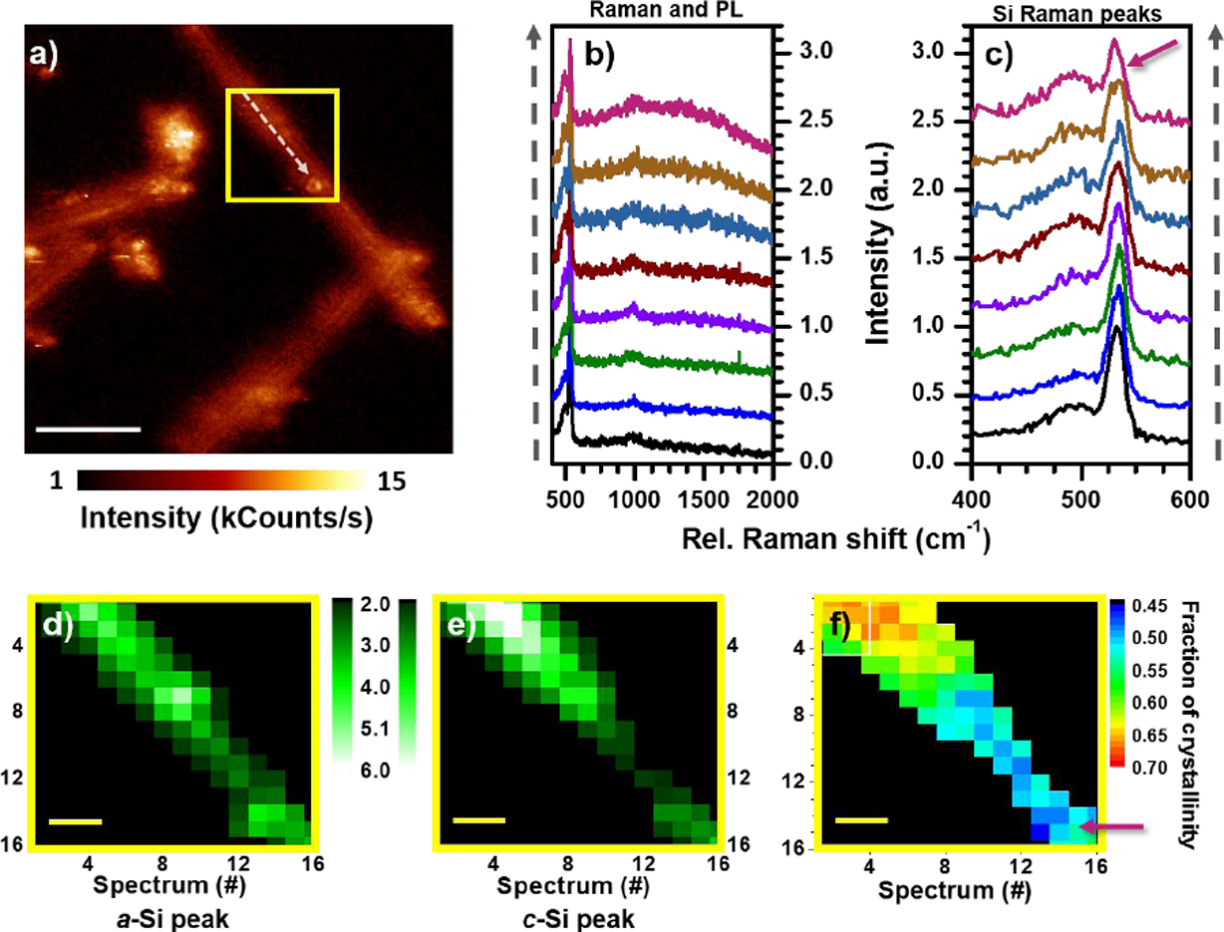
a) Confocal 20 × 20 µm^2^ image of two intersecting SiNWs on a gold substrate. The yellow square marks the region where a hyperspectral image with16 × 16 spectra was recorded. b) Raman spectra with PL background were collected along the white arrow inside the yellow square in panel a. c) The evolution in a-Si and c-Si peak intensity along the scan direction shown in panel a. Spectra are vertically shifted for clarity. d) Integrated area of the a-Si peak. e) Integrated area of the c-Si peak. f) Calculated fraction of crystallinity. The drop in the fraction of crystallinity at the right bottom corner, corresponds to the ring-shaped pattern inside the yellow square in panel a. Its corresponding spectrum and position are indicated with a pink arrow in panels c and f, respectively. The white scale bar in panel a indicates 5 μm, and the yellow scale bars are 1 μm in panels d–f.

In order to obtain a detailed map of the crystalline fraction (*f*_c_*)* along a single SiNW, 16 × 16 spectra are collected from a 5 × 5 µm^2^ area, which is marked by the yellow frame in Figure 3a. Each hyperspectral image consists of 16 × 16 spectra, resulting in a step size of 312.5 nm with a diffraction-limited laser focus of about 300 nm. In Figure 3b,c Raman spectra taken along the white arrow are shown. In Figure 3c, the Raman peaks of a-Si and c-Si were acquired with a better resolving grating with 600 lines/mm in contrast to the 150 lines/mm grating in panel b). The pink arrow in Figure 3c indicates the spectrum taken close to the ring-shaped pattern within the yellow frame in Figure 3a.

It can be clearly seen that the ratio between the intensities of a-Si and c-Si Raman peaks varies along the SiNW, mainly due to a change in the c-Si Raman intensity. To quantify this tendency, the 16 × 16 spectra are processed by peak fitting and integration as described above. The intensity distribution of the a-Si Raman peak is plotted in Figure 3d and appears to be rather constant along the silicon wire. It agrees with the TEM image in Figure 1c, where a homogenous presence of the amorphous component in the shell can be seen. In contrast, the intensity of the c-Si peak (Figure 3e) shows a clear decrease at the lower part of the SiNW. This could originate from a defect, visible as the ring-shaped pattern in Figure 3a, located at the bottom end of the yellow square. This observation is in line with the high-resolution TEM image in Figure 1c, where a migrated metal particle from the catalyst is shown. Inserting the intensity information from Figure 3d,e into Equation 1, a map of *f*_c_ along the SiNW is obtained. As shown in Figure 3f, the lowest crystalline fraction is about 0.45, and the highest is 0.70. Hence, an overview of the crystallinity of a single SiNW can be obtained at the diffraction-limited optical resolution of about 300 nm.

### Tip-enhanced Raman spectroscopy of silicon nanowires

**Figure 4:**
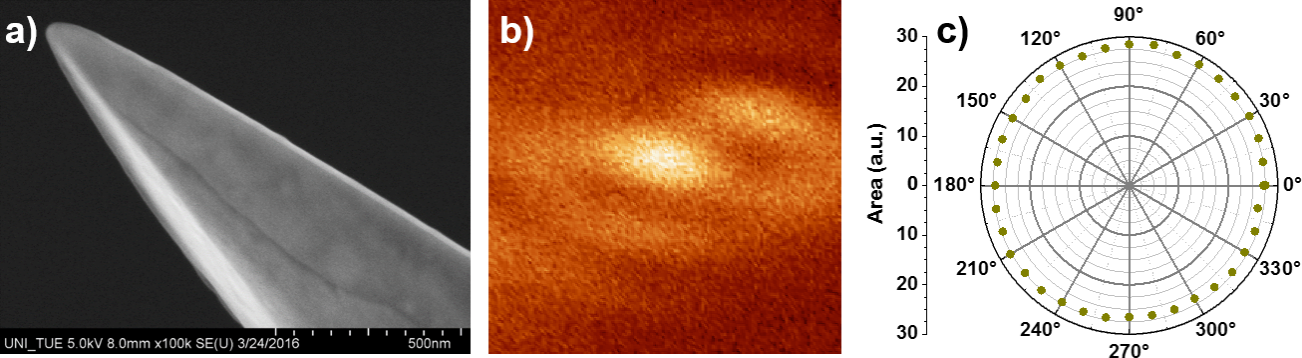
a) SEM image of the tip used. b) Optical image of the tip in focus. c) Polarization angle-resolved luminescence of the tip in focus without a sample; an almost perfectly circular emission pattern is observed.

The evolution of c-Si and a-Si Raman peaks, and thus of the fraction of crystallinity of a single SiNW, is further studied using TERS, and the emitted optical signals are analyzed using polarization angle-resolved spectroscopy. Figure 4a shows a SEM image of the tip used for the TERS measurements. Scanning the tip across the laser focus gives an Airy disc-like pattern (Figure 4b), which is due to the photoluminescence emitted from the sharp tip apex. Irregularities can arise from the slight asymmetry of the tip apex. Figure 4c shows the polarization angle-resolved optical pattern of the photoluminescence of the gold tip. We positioned a Glan–Taylor polarizer in the beam in front of the entrance slit of the spectrometer and collected spectra while rotating the polarizer. The Glan–Taylor prism only transmits the optical signal along its fast axis. Therefore, the angle-resolved polarization of the emitted signal can be obtained by rotating the prism while collecting optical spectra. The circle pattern is originating from the emission of the plasmonic oscillation along the tip shaft. This is in good agreement with the electric field distribution in the focus of a radially polarized laser beam, where the dominant field component lays out-of-plane (parallel to the tip shaft). Figure 4b,c demonstrates that the tip apex can be easily excited, which is a precondition for producing a localized near field at the tip apex.

**Figure 5:**
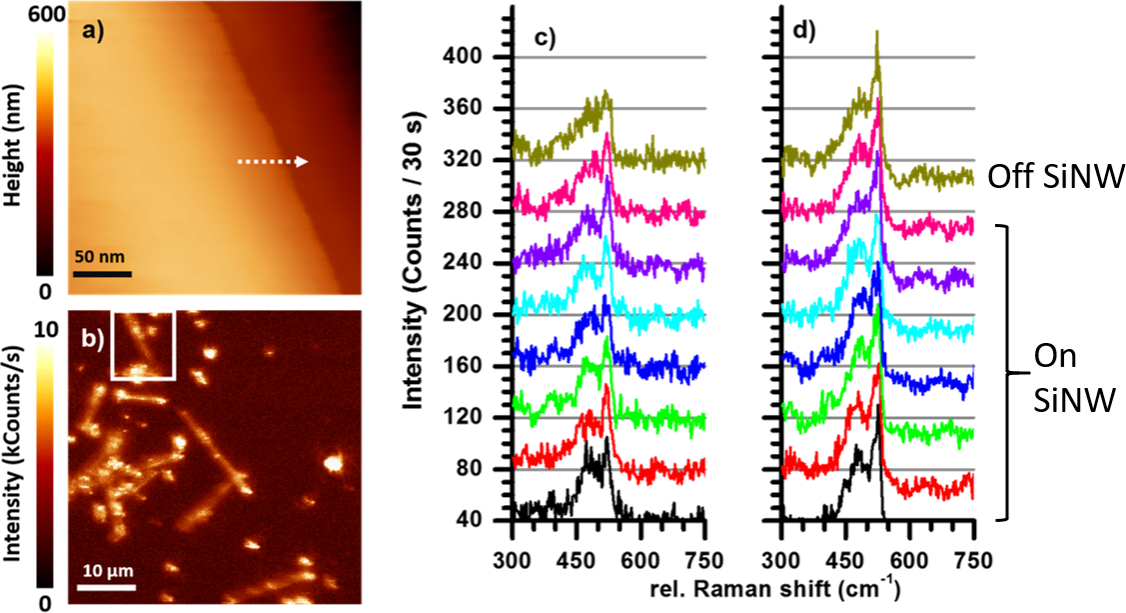
a) A shear-force scanning probe microscopy topography image (250 × 250 nm^2^) of a silicon wire edge. The white arrow indicates the range and direction along which the Raman spectra in panels c and d were recorded. b) Location of the wire indicated on the 50 × 50 µm^2^ optical image by a white square. c) TERS spectra recorded with a separation of 8 nm, vertically offset for clarity. A decreasing c-Si peak can be seeing when moving away from the edge along the arrow. d) Confocal spectra taken at exactly the same spots as the TERS spectra; the intensity ratios between the c-Si and a-Si show almost no variation. Both TERS and confocal spectra have been acquired with 170 µW excitation power and an integration time of 30 s per spectrum.

Next, we approached the sample to the tip and recorded the topography (size: 250 × 250 nm^2^) along the perimeter of a SiNW. The tip–sample distance is controlled by a shear-force feedback. For this purpose, the tip is mounted on an oscillating tuning fork, which experiences a phase shift of the oscillation upon approach. This phase shift is recorded with a lock-in amplifier and fed to a feedback loop that maintains a constant distance to the sample. The scanned SiNW perimeter is indicated in Figure 5a. Along the white arrow, there is about 250 nm height difference between the SiNW and the underlying substrate. The white square shown in the optical image in Figure 5b highlights the SiNW measured using the shear-force scanning function of our custom-built TERS setup. Eight TERS spectra were taken along the white arrow in Figure 5a over a length of 64 nm. The distance between the two sequential spectra is 8 nm. The spectra were acquired from bottom to top, with the black spectrum on the SiNW and the yellow-green one on the underlying substrate. Notably, although a clear decrease in c-Si intensity is observed across the perimeter of the SiNW, the a-Si Raman peak intensity remains nearly constant and indicates the crystalline state of the SiNW shell. The Raman intensity evolutions of the c-Si and a-Si are shown in Figure S1a (Supporting Information File 1). We clearly show a decrease in the fraction of crystallinity from the center to the perimeter of the SiNW, which can be resolved with the optical resolution of 8 nm. These observations agree well with the morphology revealed by high-resolution TEM images in Figure 1c.

As a reference measurement, an equal number of far-field spectra (normal Raman spectra) are taken from the same sample positions (Figure 5d). Even between the SiNW and the underlying substrate, the decrease in the c-Si Raman peak is barely visible. This can be explained by the excitation area of the confocal laser focus, which is at least one order of magnitude larger than that in the TERS experiments. Therefore, only a marginal difference between two spectra of 8 nm apart is visible, even when the sampling point is not on the SiNW anymore (“off SiNW” spectrum, Figure 5d, and Figure S1b in Supporting Information File 1). Beeman et al. [22] suggested to use the root mean square bond-angle distortion ΔΘ to evaluate the crystalline and amorphous fractions of Si, which can be calculated using the full width half maximum (FWHM, in cm^−1^) of the one-phonon Raman peak of Si at about 520 cm^−1^ with the equation:

[2] $$\text{FWHM}=\frac{\left(15+6\Delta \Theta \right)}{2}.$$

In c-Si with cubic diamond structure, ΔΘ has a value of 0°, whereas in a-Si films ΔΘ was experimentally determined to be in the range of 7.7° ≤ ΔΘ ≤ 10.5°. In our experiments we calculated ΔΘ_avg,n-f_ = 9.2° and ΔΘ_avg,conf_ = 8.3° based on the near-field and confocal spectra, respectively. The slightly higher value of ΔΘ determined with TERS could be due to the nanometer-sized penetration depth of the near field excited at the tip apex, in contrast to the far-field laser focus. Since the SiNWs contain a c-Si core and an a-Si shell (Figure 1), the shell can be excited stronger by the evanescent electromagnetic near field at the tip apex.

**Figure 6:**
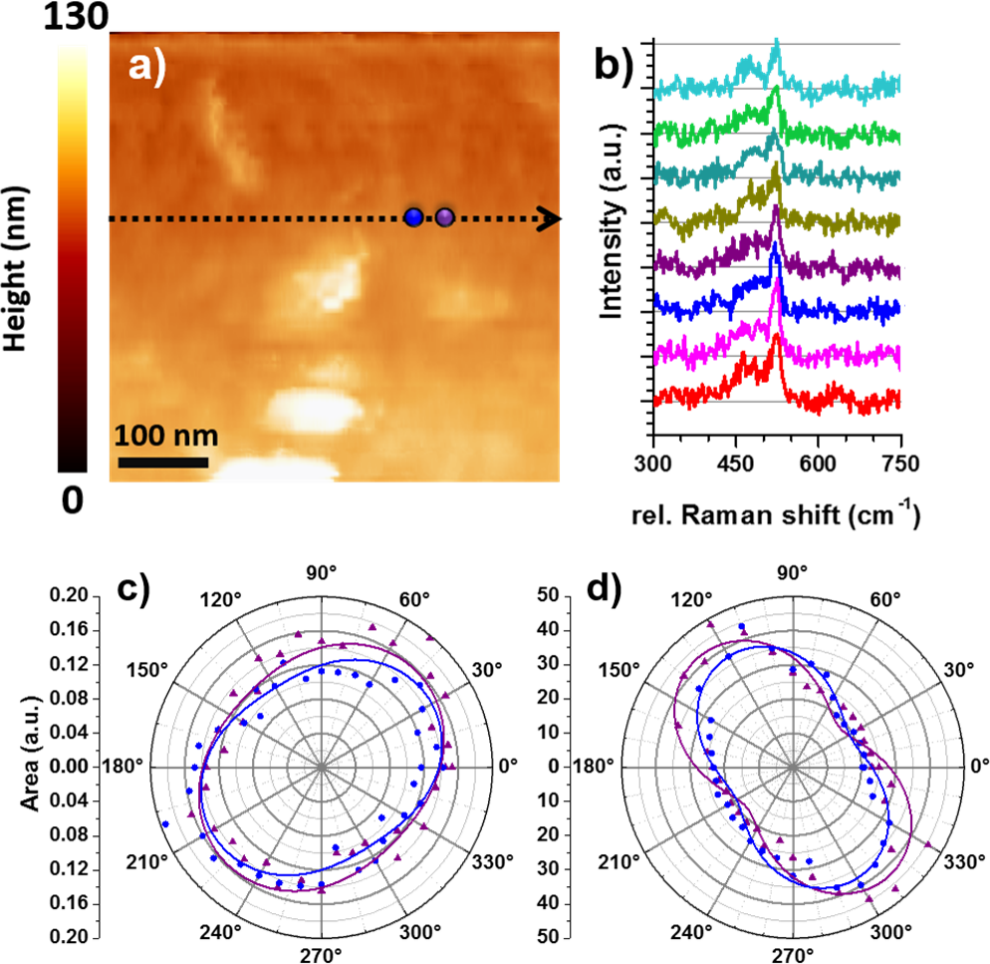
a) Topographical image of the top of a SiNW. The dashed arrow shows the region in which the 32 Raman spectra were recorded. The blue and the purple dot indicate the positions where the polarization angle-resolved measurements where performed. b) Eight of the collected Raman spectra along the white arrow in panel a showing varying c-Si Raman peak intensities. A detailed analysis about the intensity evolution of the a-Si and c-Si Raman peaks is shown in Figure S2 (Supporting Information File 1). c) Polarization angle-resolved Raman scattering of the c-Si Raman peak as a function of the rotation angle of the Glan–Taylor polarizer. The triangles and circles are the original data, whilst the closed lines are to guide the eyes. d) Polarization angle-resolved photoluminescence patterns collected from the same sample positions, indicated in panel a.

The highly improved optical resolution achieved with TERS depends strongly on the tip–sample interaction. When the sample is positioned within close proximity of the excited tip apex, the substrate material gains influence via the coupling between the oscillation field in the excited tip and its mirror image in the substrate. We have shown in a previous theoretical work that the permittivity of the tip and the substrate influences the near-field enhancement at the tip apex significantly [33]. In the next set of experiments, we combined angle-resolved polarization measurements with TERS to investigate the effects of tip–sample interactions on the optical signals. In Figure 6a the topography of a SiNW surface is shown. Along the dashed arrow, 32 spectra were recorded, and eight of them are plotted in Figure 6b. Although all spectra are taken on the surface of one SiNW, the intensity ratio differences between the c-Si and a-Si Raman peaks are clearly visible (Figure S2, Supporting Information File 1), which can be attributed to the variations of the local crystallinity within the SiNW. Furthermore, polarization angle-resolved emission measurements are performed at two adjacent positions 30 nm apart, marked by the blue and the purple point in Figure 6a. The integrated area of the c-Si Raman peak is plotted as a function of the rotation angle of the fast axis of the Glan–Taylor polarizer. The measured polarization angle-resolved emission patterns from the two sample positions are rather similar, indicating similar polarization states of the Raman scatterings. Interestingly, when plotting the photoluminescence signal as a function of the rotation angle of the Glan–Taylor polarizer, as shown in Figure 6d, the patterns are distinctly different from those in Figure 6c. Note that the Raman intensity and the photoluminescence intensity used for the plots in Figure 6c and Figure 6d are derived from the same spectrum. Therefore, potential artefacts induced by any instrument operation errors can be excluded. Furthermore, upon approaching the gold tip to the SiNW, the angle-resolved polarization pattern changes from a circular pattern (Figure 4c) to a more structured shape in Figure 6d. It is likely that the high refractive index of the SiNW has a certain impact on the polarization of the photoluminescence emitted from the narrow gap between the gold tip and the SiNW. A more quantitative investigation of the polarization angle-resolved emission patterns in Figure 6 will be further pursued. The results shown in Figure 4 and Figure 6 demonstrate that it is possible to combine the polarization angle-resolved experiments with a TERS setup, which has been rarely pursued so far. The successful combination of both techniques is promising for developing new strategies to resolve the structural properties at the sub-10 nanometer scale, based on the polarization properties of the optical signals, demonstrated here for c-Si and a-Si using a core–shell SiNW.

## Conclusion

Local structural properties, such as changes in the fraction of crystallinity of a c-Si/a-Si core–shell nanowire are characterized confocally at an optical resolution of about 300 nm. Applying tip-enhanced Raman spectroscopy, we show that variations of the intensity ratio between the crystalline Si and amorphous Si Raman peaks at sample positions as close as eight nanometers can be revealed. Furthermore, the polarizations of Raman scattering and photoluminescence signals locally emitted from a single SiNW are explored by combining polarization angle-resolved emission spectroscopy with tip-enhanced optical spectroscopy. Our work demonstrates the high potential of resolving local structural properties of nanomaterials, here demonstrated for silicon, at the sub-10 nanometer scale using tip-enhanced Raman techniques. TEM investigations are in line with TERS results, which supports the idea that TERS can be used as a micro/nano-structure characterization technique.

## Experimental

Core–shell SiNWs were synthesized in two steps. At first, SiNWs were grown by utilizing the VLS growth mechanism [13] using dewetted Pt thin films as the growth catalyst [29] at a growth temperature of 720 °C, which yields a certain SiNW diameter distribution. VLS nanowire growth is carried out in a quartz tube furnace with a precursor gas mixture of H_2_ (270 sccm) and SiH_4_ (30 sccm), at a pressure of 100 mbar. Silicon shells are grown at a temperature of 520 °C with a gas mixture of H_2_ (10 sccm) and SiH_4_ (100 sccm), at a pressure of 100 mbar. The silicon shell growth rate under these conditions is in the range of 1 nm/min and yields a thickness of approximately 25 nm. To make confocal Raman and TERS investigations of individual nanowires, SiNWs are released from their original growth substrate by ultra-sonicating the growth substrate in purified water. For Raman and TERS investigations, nanowires are deposited on gold-coated silicon wafers as carrier substrates. For transmission electron microscopy (TEM), nanowires are deposited on copper TEM grids with lacey carbon meshes.

Using a custom-built setup with a parabolic mirror (NA: 0.998) [34], we achieve a diffraction-limited confocal resolution by illumination with a radially polarized laser beam [35]. With a 636.3 nm diode laser operating in continuous-wave mode we obtain a focus diameter of roughly 300 nm [36]. To further increase the resolution a chemically etched gold tip, attached to a tuning fork, is brought into the focus [37–41]. A plasmonic oscillation is generated at the tip apex by the excitation with a radially polarized laser beam along the tip axis. Here, the local field intensity is greatly increased leading to a strong local near field confined at the tip apex. This gives rise to the enhanced sensitivity of tip-enhanced Raman spectroscopy (TERS). TERS combined with scanning probe microscopy (SPM) also allows for the collection of correlated topography and optical images [42–43]. For polarization angle-resolved emission measurements, a Glan–Taylor prism as polarization filter is positioned in front of the entrance slit of the spectrometer.

## Supporting Information

File 1 Additional experimental data.
