# Supplementary material for: Revealing the local crystallinity of single silicon core–shell nanowires using tip-enhanced Raman spectroscopy
Source: Beilstein J Nanotechnol. 2020 Jul 31;11:1147–56. doi: 10.3762/bjnano.11.99 (PMC7404174; doi:10.3762/bjnano.11.99)
Supplement: File 1 — Additional experimental data. [file Beilstein_J_Nanotechnol-11-1147-s001.pdf]

BEILSTEIN JOURNAL OF NANOTECHNOLOGY

Supporting Information

for

Revealing the local crystallinity of single silicon core–shell nanowires using tip-enhanced Raman spectroscopy

Marius van den Berg, Ardeshir Moeinian, Arne Kobald, Yu-Ting Chen, Anke Horneber, Steffen Strehle, Alfred J. Meixner and Dai Zhang

Beilstein J. Nanotechnol. **2020,** *11,* 1147–1156. doi:10.3762/bjnano.11.99

Additional experimental data

License and Terms: This is a supporting information file under the terms of the Creative Commons Attribution License (http://creativecommons.org/licenses/by/4.0). Please note that the reuse, redistribution and reproduction in particular requires that the authors and source are credited.
The license is subject to the *Beilstein Journal of Nanotechnology* terms and conditions: (https://www.beilstein-journals.org/bjnano)

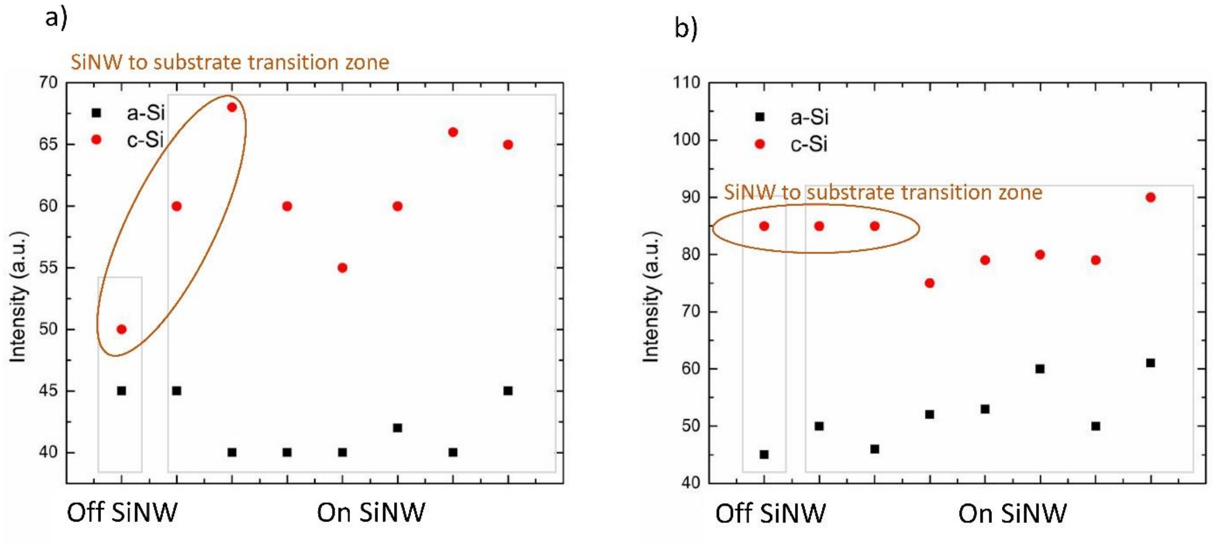

Figure S1: a) and b) are the intensity evolutions of the a-Si and c-Si Raman peaks shown in Figure 5c and Figure 5d, respectively. In the TERS measurements shown in Figure S1a, the a-Si Raman peak intensity stays nearly constant indicating the crystal state of the SiNW shell; while the c-Si intensity varies across the perimeter of the SiNW. In the confocal measurements shown in Figure S1b, the variations in the c-Si intensity, for example at the transition zone, are smaller than those from the TERS measurements due to the limited optical resolution.

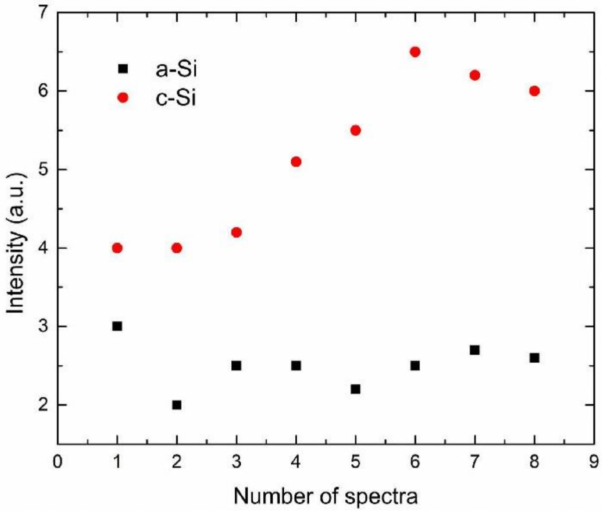

Figure S2: The intensity evolution of the a-Si and c-Si Raman peaks shown in Figure 6b. The 8th data points are derived from the red spectrum in Figure 6b; while the 1st data points are derived from the light blue spectrum. Here, the variation in the local crystallinity can be seen from the evolution in the c-Si Raman intensity.
